# Supplementary material for: Using Steady-State Kinetics to Quantitate Substrate Selectivity and Specificity: A Case Study with Two Human Transaminases
Source: Molecules. 2022 Feb 18;27(4):1398. doi: 10.3390/molecules27041398 (PMC8875635; doi:10.3390/molecules27041398)
Supplement: Supplementary file 1 [file molecules-27-01398-s001.zip › molecules-1585601-supplementary.pdf]

molecules

MDPI

Supplementary materials to:

Using Steady-State Kinetics to Quantitate Substrate Selectivity and Specificity: A Case Study with Two Human Transaminases

Alessio Peracchi [1,*] and Eugenia Polverini [2]

1 Department of Chemistry, Life Sciences and Environmental Sustainability, University of Parma, 43124 Parma, Italy
2 Department of Mathematical, Physical and Computer Sciences, University of Parma, 43124 Parma, Italy; eugenia.polverini@unipr.it
* Correspondence: alessio.peracchi@unipr.it

Supplementary Tables

Table S1. Literature data on enzyme discrimination towards alternative substrates in which a carboxylate group is replaced by an amido group (-COO- vs –CONH2).

Abbreviation	Enzyme[1]	Preferred Substrate	Alternative Substrate	D	Ref.
		Aspartate	***Asparagine***		
LDOX *S. tok*	L-aspartate oxidase from *Sulfolobus tokodaii*	V_{max}/K_M = 0.754 U mg^{-1} mM^{-1}	V_{max}/K_M = 0.012 mM^{-1} s^{-1}	62.8	[1]
LDOX *P. put*	L-aspartate oxidase from *Pseudomonas putida*	V_{max}/K_M=4.69 U mg^{-1} mM^{-1}	V_{max}/K_M =3.6 U mg^{-1} mM^{-1}	1.3	[2]
ATC *E. coli*	Aspartate transcarbamoylase from *E. coli*	V_{max}/S_{05} = 23,6 U mg^{-1} mM^{-1}	V_{max}/S_{05} = 0.22 U mg^{-1} mM^{-1}	108	[3]
AK *E. coli*	Aspartokinase from *E. coli*	k_{cat}/K_M = 167 A.U. mM^{-1}	k_{cat}/K_M = 2.8 A.U. mM^{-1}	59.3	[4]
LAOX *B. fas*	L-Amino acid oxidase from *Bungarus fasciatus*	k_{cat}/K_M = 1187.25 mM^{-1} s^{-1}	k_{cat}/K_M = 4.41 mM^{-1} s^{-1}	269.2	[5]
GOT1 *H. sap*	Aspartate aminotransferase from *Homo sapiens*	k_{cat}/K_M = 63 mM^{-1} s^{-1}	k_{cat}/K_M = 3.6 × 10^{-5} mM^{-1} s^{-1}	1.07 × 10^{6}	This work
DDOX *O. hep*	D-Aspartate oxidase from *Octopus hepatopancreas*	k_{cat}/K_M = 1.58 mM^{-1} s^{-1}	k_{cat}/K_M = 0.005 mM^{-1} s^{-1}	316	[6]
DDOX *B. tau*	D-Aspartate oxidase from *Bos taurus*	k_{cat}/K_M = 22.2 A.U. mM^{-1}	k_{cat}/K_M = 0.3 A.U. mM^{-1}	74	[7]
		Glutamate	***Glutamine***		
LAOX *B. fas*	L-Amino acid oxidase from *B. fasciatus*	k_{cat}/K_M = 548.95 mM^{-1}s^{-1}	k_{cat}/K_M = 73.19 mM^{-1}s^{-1}	7.5	[5]
DDOX *T. dup*	D-Aspartate oxidase from *Thermomyces dupontii*	k_{cat}/K_M = 100 mM^{-1} s^{-1}	k_{cat}/K_M = 1.33 mM^{-1} s^{-1}	75.2	[8]
AAT *E. coli*	Aspartate aminotransferase from *E. coli*	k_{cat}/K_M = 4,79 mM^{-1} s^{-1}	k_{cat}/K_M = 0.00036 mM^{-1} s^{-1}	13232	[9]
GOT1 *H. sap*	Aspartate aminotransferase from *H. sapiens*	k_{cat}/K_M = 63 mM^{-1} s^{-1}	k_{cat}/K_M = 3.6 × 10^{-5} mM^{-1} s^{-1}	750,000	This work
GPT *H. sap*	Alanine aminotransferase from *H. sapiens*	k_{cat}/K_M = 12 mM^{-1} s^{-1}	k_{cat}/K_M = 0.001 mM^{-1} s^{-1}	12,000	This work
		Arginine	***Argininamide***		
RASE *R. nor*	Arginase from *Rattus norvegicus*	k_{cat}/K_M = 2600 mM^{-1} s^{-1}	k_{cat}/K_M = 15 mM^{-1} s^{-1}	173	[10]
		Tyrosine	***Tyrosinamide***		
PST *E. gra*	Phenol sulfotransferase from *Euglena gracilis*	V_{max}/K_M = 1.67 U mg^{-1} mM^{-1}	V_{max}/K_M = 0.21 U mg^{-1} mM^{-1}	8	[11]
		Tryptophan	***Tryptophanamide***		

WOX *C. vio*	L-tryptophan 2,3 oxidase from *Chromobacterium violaceum*	k_{cat}/K_M = 7000 mM^{-1} s^{-1}	k_{cat}/K_M = 4000 mM^{-1} s^{-1}	1.75	[12]
		Histidine	***Histidinamide***		
OVOA *E. tas*	L-cisteinyl L-Histidinyl sulfoxide syntase from *Erwinia tasmaniensis*	k_{cat}/K_M = 10 mM^{-1} s^{-1}	k_{cat}/K_M = 1.8 mM^{-1} s^{-1}	5.6	[13]
		Nicotinate	***Nicotinamide***		
NMAT *H. sap*	Nicotinamide mononucleotide adenylyltransferase 2 from *H. sapiens*	k_{cat}/K_M = 479 mM^{-1} s^{-1}	k_{cat}/K_M = 411 mM^{-1} s^{-1}	1.15	[14]
NMAT *B. sub*	Nicotinate mononucleotide adenyltransferase from *Bacillus subtilis*	V_{max}/K_M = 258 U mg^{-1} mM^{-1}	V_{max}/K_M = 0.02 U mg^{-1} mM^{-1}	12,900	[15]
		Glutathione disulfide	***Glutathione amide disulfide***		
GR *H. sap*	Glutathione reductase from *H. sapiens*	k_{cat}/K_M = 2360 mM^{-1} s^{-1}	k_{cat}/K_M = 27 mM^{-1} s^{-1}	87.8	[16]

[1] Data for the present table were obtained as described in the Methods, by defining first a list of metabolite pairs showing the difference of interest (e.g., L-aspartate/L-glutamine; nicotinic acid/nicotinamide etc.). Then, for each couples of substrates, the literature was thoroughly searched (beginning with the BRENDA repository of enzyme data [17]) to find enzymes whose reaction with both substrates had been comparatively analyzed in the same study. Notably, for some enzymes, it was reported that an alternative substrate bearing the amido group was completely unreactive. For example Ueno and coworkers [18] reported that a *Lactobacillus brevis* glutamate decarboxylase exhibited no activity against glutamine. However, this does not mean that the discrimination index of the enzyme was infinite, but – most likely - that reaction with glutamine was too slow to be detected under the conditions in which the authors conducted their assays.

Table S2. Literature data on enzyme discrimination towards alternative substrates in which a carboxylate group is replaced by an H atom ($-COO^-$ vs –H).

Abbreviation	Enzyme	Preferred Substrate	Alternative Substrate	D	Ref.
		**Aspartate**	**Alanine**		
AAT *E coli*	Aspartate aminotransferase from *E. coli*	k_{cat}/K_M = 130 mM^{-1} s^{-1}	k_{cat}/K_M = 0.51 mM^{-1} s^{-1}	255	[19]
GOT1 *H. sap*	Aspartate aminotransferase from *H. sapiens*	k_{cat}/K_M = 63 mM^{-1} s^{-1}	k_{cat}/K_M = 4.3 × 10^{-5} mM^{-1} s^{-1}	1.47 × 10^6	This study
DDOX *V. hum*	D-aspartate oxidase from *Vanrija humicola*	k_{cat}/K_M = 21.5 mM^{-1} s^{-1}	k_{cat}/K_M = 0.0022 mM^{-1} s^{-1}	10,070	[20]
		**Glutamate**	**2-aminobutyrate**		
BCAT *Th. spp*	Branched-chain amino acid aminotransferase from *Thermus sp.*	k_{cat}/K_M = 36 mM^{-1} s^{-1}	k_{cat}/K_M = 11 mM^{-1} s^{-1}	3.3	[21]
		**Arginine**	**Agmatine**		
RASE *R. nor*	Arginase from *R. norvegicus*	k_{cat}/K_M = 2600 mM^{-1} s^{-1}	k_{cat}/K_M = 0.048 mM^{-1} s^{-1}	54,200	[10]
PPRD *P. ging*	Peptidylarginine deiminase from *Porphyromonas gingivalis*	k_{cat}/K_M = 0.19 mM^{-1} s^{-1}	k_{cat}/K_M = 2.3 ×10^{-4} mM^{-1} s^{-1}	826	[22]
		**Orotate**	**Uracil**		
OPT *S. typh*	Orotate phosphoribosyltransferase from *Salmonella typhimurium*	k_{cat}/K_M = 980 mM^{-1} s^{-1}	k_{cat}/K_M = 6 × 10^{-4} mM^{-1} s^{-1}	1.63 × 10^6	[23]
		**Homoserine**	**3-amino-1-propanol**		
HSTA *H. inf*	Homoserine transacetylase from *Haemophilus influenzae*	k_{cat}/K_M = 710 mM^{-1} s^{-1}	k_{cat}/K_M = 0.046 mM^{-1} s^{-1}	15,400	[24]
		**Histidine**	**Histamine**		
OVOA *E. tas*	L-cisteinyl L-Histidinyl sulfoxide syntase from *Erwinia tasmaniensis*	k_{cat}/K_M = 10 mM^{-1} s^{-1}	k_{cat}/K_M = 1.6 mM^{-1} s^{-1}	6.25	[13]
		**Malonyl-CoA**	**Acetyl-CoA**		
MAT1 *V. hyb*	3-O-glucoside-6″-O-malonyltransferases from *Verbena hybrida*	k_{cat}/K_M = 930 mM^{-1} s^{-1}	k_{cat}/K_M = 0.9 mM^{-1} s^{-1}	1033	[25]
MAT1 *L. pur.*	3-O-glucoside-6″-O-malonyltransferases from *Lamium purpureum*	k_{cat}/K_M = 360 mM^{-1} s^{-1}	k_{cat}/K_M = 0.8 mM^{-1} s^{-1}	450	[25]

[1] Data for the present table were obtained as described in the Methods and in the legend of Table S1.

Table S3. Literature data on enzyme discrimination: cases in which a linear preferred substrate is compared with an alternative substrate whose extremities are preserved, but whose length is shorter by one methylene group (–CH_2–).

Abbreviation	Enzyme	Preferred substrate	Alternative substrate	D	Ref.
		**Leucine**	**Valine**		
LEDH *T. int*	Leucine dehydrogenase from *Thermoatinomyces intermedium*	V_{max}/K_M = 60 AU mM^{-1}	V_{max}/K_M = 44,79 AU mM^{-1}	1.33	[26]
BCAT *M. aeo*	Branched-chain amino acid aminotransferase from *Methanococcus aeolicus*	V_{max}/K_M = 1.36 U/mg mM^{-1}	V_{max}/K_M = 0.39 U/mg mM^{-1}	3.56	[27]
BCAT *S. lyco*	Branched-chain amino acid aminotransferase from *Solanum lycopersicum*	k_{cat}/K_M = 69.82 mM^{-1} s^{-1}	k_{cat}/K_M = 50.5 mM^{-1} s^{-1}	1.38	[28]
LTRS *E. coli*	Leucyl-tRNA sinthetase from *E.coli*			3200	[29]
		**Glutamate**	**Aspartate**		
EDEC *S. scr*	Glutamate decarboxylase from *Sus scrofa*	k_{cat}/K_M = 12 mM^{-1} s^{-1}	k_{cat}/K_M = 0,31 mM^{-1} s^{-1}	38.7	[30]
GPT *H. sap*	Alanine aminotransferase from *H. sapiens*	k_{cat}/K_M = 12 mM^{-1} s^{-1}	k_{cat}/K_M = 0.0057 mM^{-1} s^{-1}	2150	This work
		**Glutamine**	**Asparagine**		
GLNS *B. pas*	Glutaminase from *Bacillus pasteurii*	V_{max}/K_M = 10.5 AU mM^{-1}	V_{max}/K_M = 0.86 AU mM^{-1}	12.3	[31]
GLNS *S. can*	Glutaminase from *Steptomyces canarius*	5.81 × 10^{-3} mM^{-1} s^{-1}	4.96 × 10^{-3} mM^{-1} s^{-1}	1.17	[32]
GLNS *S. can*	Glutaminase from *Pseudomonas sp.*	20,005	12752.55	1.56	[33]
		**Adenosyl-Homocysteine**	**Adenosyl-Cysteine**		
AHOC *L. lut*	Adenosyl-homocysteinase from *Lupins lutenus*	k_{cat}/K_M = 60,000 $M^{-1}s^{-1}$	k_{cat}/K_M = 0.9 $M^{-1}s^{-1}$	66,700	[34]
		**Homocysteine**	**Cysteine**		
CBS *L. pla*	Cystathionine ◎-sinthase from *Lactobacillus plantarum*	k_{cat}/K_M = 14 $mM^{-1}s^{-1}$	k_{cat}/K_M = 1.3 $mM^{-1}s^{-1}$	10.76	[35]
CBS *H. sap*	Cystathionine ◎-sinthase from *H. sapiens*	k_{cat}/K_M = 1.93 $mM^{-1}s^{-1}$	k_{cat}/K_M= 0.53 $mM^{-1}s^{-1}$	3.65	[36]
HCDS *A. fum*	Homocysteine desulfhydrase from *Aspergillus funigatus*	k_{cat}/K_M = 0.56 $mM^{-1}s^{-1}$	k_{cat}/K_M = 0.155 $mM^{-1}s^{-1}$	3.6	[37]
		**Lysine**	**Ornithine**		
LKOX *T. vir*	L-Lysine oxidase from *Trichoderma viride*	V_{max}/K_M = 2520 AU/mg mM^{-1}	V_{max}/K_M = 417 AU mM^{-1}	6.04	[38]
KMOX *P. flu*	Lysine 2-monooxygenase from *Pseudomonas fluorescens*	V_{max}/K_M = 83.87 U/mg mM^{-1}	V_{max}/K_M = 0.31 U/mg mM^{-1}	270.5	[39]
KRAC *O. oen*	Lysine racemase from *Oenococcus oeni*	k_{cat}/K_M = 19 min^{-1} mM^{-1}	k_{cat}/K_M = 4.1 min^{-1} mM^{-1}	4.63	[40]

[1] Data for the present table were obtained as described in the Methods and in the legend of Table S1.

Supplementary Figures

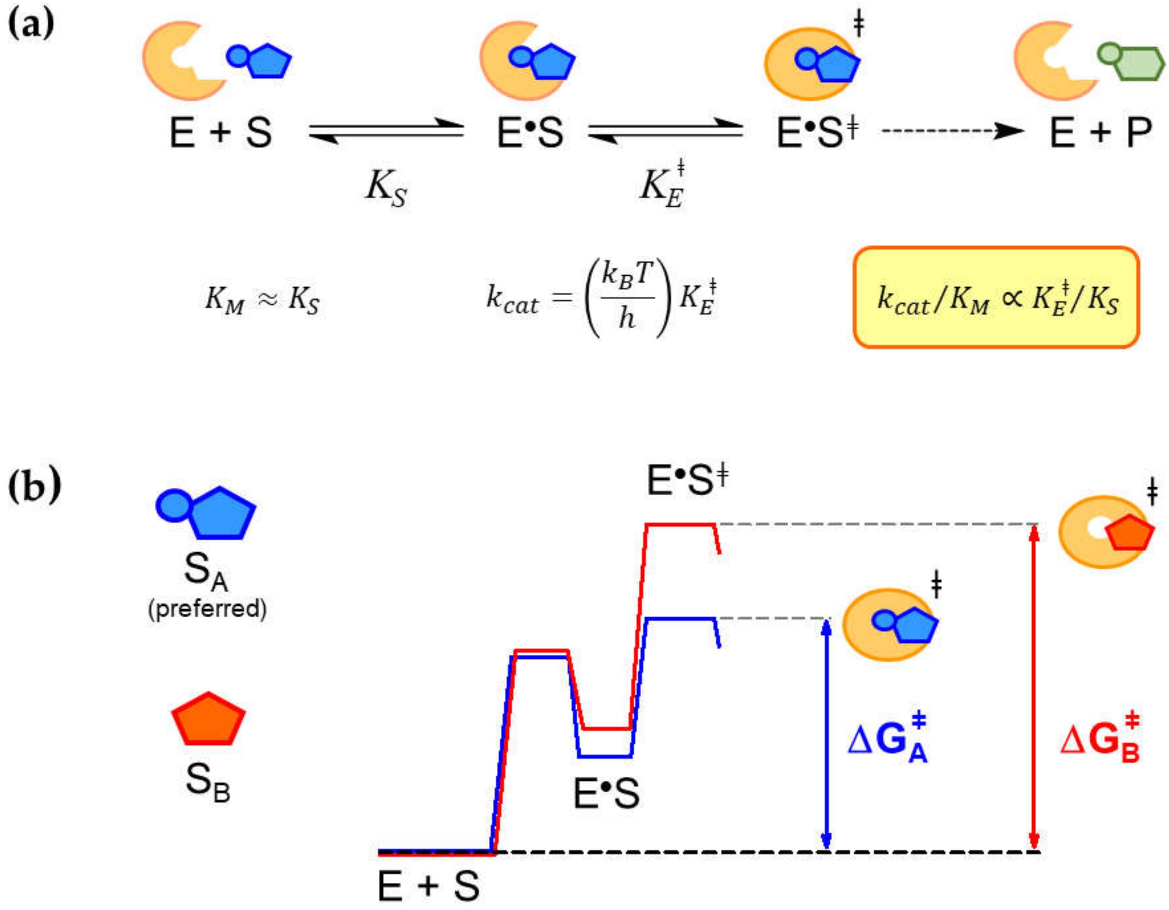

Figure S1. "Canonical" and "rotated conformations of the different substrates docked in the GOT1 active site, with the corresponding clustering on the left (rmsd threshold = 2 Å). Only Lys259 and the arginine residues are shown for clarity, while the α-carboxylate of the substrate is circled in orange as a reference. Red clusters correspond to the canonical conformation, blue clusters to the rotated one. The calculated binding energy for each conformation is also reported.

$$k_{cat} = \left(\frac{k_B T}{h}\right) K_E^‡ \quad \text{(I)}$$

where k_B is Boltzmann's constant, T is the absolute temperature and h is Planck's contant. Hence:

$$k_{cat}/K_M \approx \left(\frac{k_B T}{h}\right) K_E^‡/K_S \quad \text{(II)}$$

(b) Free energy diagram for the kinetic model shown in Panel a. As it can be derived from the equation above and from the basic thermodynamic relationships, the free-energy difference, $\Delta G^‡$, between the free reactants (E + S) and the transition state complex is proportional to the negative logarithm of k_{cat}/K_M.

$$\Delta G^‡ = -RTln(K_E^‡/K_S) = -RTln(k_{cat}/K_M) + RTln\left(\frac{k_B T}{h}\right) \quad \text{(III)}$$

Differences in k_{cat}/K_M between alternative substrates will reflect their differential binding interactions in the transition state. Interactions that form in the initial E•S complex (and are subsequently maintained) as well as interactions that are exclusively formed in the enzyme-bound transition state stabilize the transition state itself (with respect to the free enzyme and free substrate). Loss of such interactions (for a suboptimal substrate) will destabilize the transition state and lower k_{cat}/K_M. In the hypothetical example presented here, the alternative substrate B forms a less stable E•S complex as compared to the preferred substrate A (higher free energy, hence higher K_M) and an even less stable transition state (lower k_{cat}).

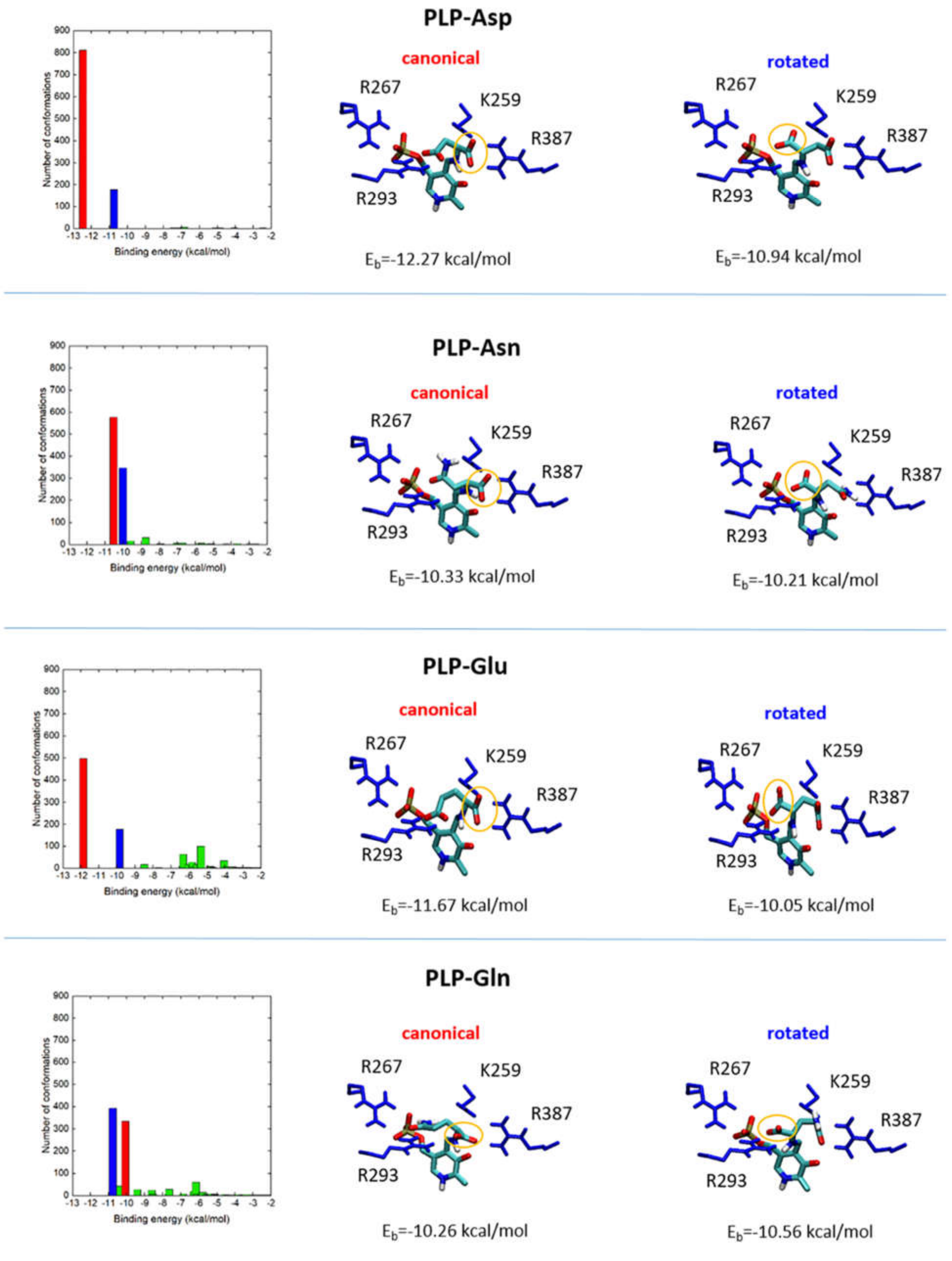

Figure S2. "Canonical" and "rotated conformations of the different substrates docked in the GOT1 active site, with the corresponding clustering on the left (rmsd threshold = 2 Å). Only Lys259 and the arginine residues are shown for clarity, while the α-carboxylate of the substrate is circled in orange as a reference. Red clusters correspond to the canonical conformation, blue clusters to the rotated one. The calculated binding energy for each conformation is also reported.

```
GOT1  13                 MQPVLVF KLTADFRE-- ---------D PDPRKVNLGV
GTP   19    KVLTLDGMNP RVRRVEYAVR GPIVQRALEL EQELRQGVKK PFTEVIRANI
                               *      .                 *    .   .

GOT1  39    GAYRTDDCHP WVLPVVKKVE QKIANDN--- ---------- ----------
GTP   69    GDAQAM--GQ RPITFLRQVL ALCVNPDLLS SPNFPDDAKK RAERILQACG
            *  ..      . .  .. *      * .

GOT1  66    -SLNHEYLPI LGLAEFRSCA SRLALGDDSP AL-KEKRVGG VQSLGGTGAL
GTP   117   GHSLGAYSVS -SGIQLIRE- DVARYIERRD GGIPADPNNV FLSTGASDAI
                  *     .  ..           .    .            * *.. *.

GOT1  114   RIGADFLARW YNGTNNKNTP VYVSSPTWEN HNAVFSAAGF KDIRSYRYWD
GTP   165   VTVLKLLVAG E---GHTRTG VLIPIPQYPL YSATLAELGA -VQVDYYLDE
                 .*         .  *  * .. *      .* ..  *       *   .

GOT1  164   AEKRGLDLQG FLNDLEN--- ----APEFSI VVLHACAHNP TGIDPTPEQW
GTP   211   --ERAWA--L DVAELHRALG QARDHCRPRA LCVI-NPGNP TGQVQTRECI
               *.       . .*                 . .   . ** **   * *

GOT1  207   KQIASVMKHR FLFPFFDSAY QGFASGNLER DAWAIR-YFV S------EGF
GTP   256   EAVIRFAFEE RLFLLADEVY QDNVYAAG-S QFHSFKKVLM EMGPPYAGQQ
              .         ** . *  * *    .     .  ...  ..

GOT1  250   EFFCAQSFSK NF-GLYNERV GNLTVVGKEP ESILQVLSQM EKIVRITWSN
GTP   305   ELASFHSTSK GYMGECGFRG GYVEVVNMDA ----AVQQQM LKL--MSVRL
            *.   .* **  . *    *  * . ** ...      *  **  *.  ..

GOT1  299   PP-AQGARIV ASTLSNPELF EEWTG----- ---NVKTMAD RILTMRSELR
GTP   349   CPPVPGQALL D----LVVSP PAPTDPSFAQ FQAEKQAVLA ELAAKAKLTE
             *   *  ..               *          .  ..    . ..

GOT1  340   ARLEALKTPG TWNHITDQIG MFSFTGLNPK QVE------- ----------
GTP   395   QVFNE---AP GISCNPVQGA MYSFPRVQLP PRAVERAQEL GLAPDMFFCL
              ..    .    .    * . *.**  .

GOT1  373   YLVNEKHIYL LPSG------ -----RINVS GLTTKNLDYV ATSIHEAVTK
GTP   442   RLLEETGICV VPGSGFGQRE GTYHFRMTIL PPLE-KLRLL LEKLSRFHAK
             *..*  * . .*..            *. .       .*  .    .    .*

GOT1  412   IAENLYFQ -
GTP   491   FTLEYS-
            .. .
```

Figure S3. Structural alignment of the human GOT1 and GPT enzymes. The binding site residues are shown in bold red: the charged ones are highlighted in yellow; the lysine involved in the proton transfer is highlighted in light gray; the residues belonging to the other monomer in cyan. Finally, the tyrosine that in GPT interacts with the substrate sidechain is highlighted in green.

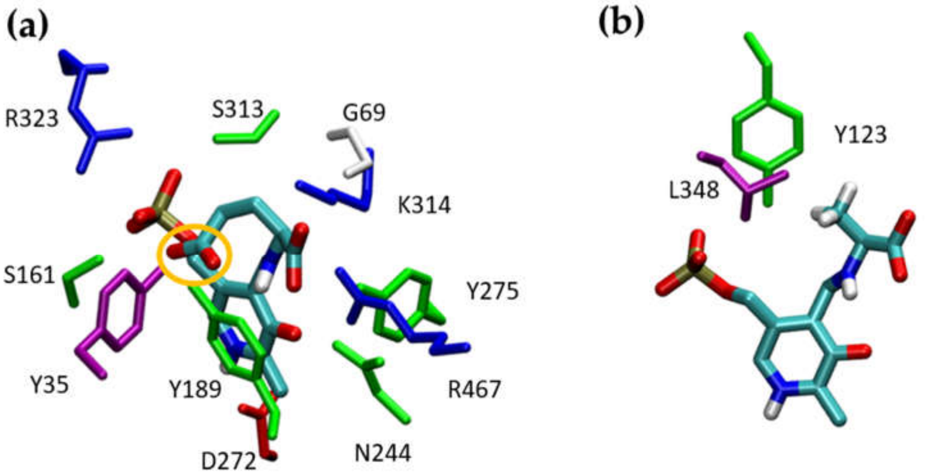

Figure S4. Active site residues of the GPT model. (a) Residues belonging to the first chain (monomer B). The PLP-glutamate Schiff base (in the canonical orientation) was inserted by superimposition with the GOT1 model. The sidechain carboxylate is circled in orange as a reference. Tyr35, that appears to functionally substitute Arg293 of GOT1, is shown in purple. Lys314, which is involved in catalytic proton transfer, points towards the α-carbon of the substrate. (b) Residues belonging to the other monomer (monomer A). In this case, the PLP-alanine adduct is shown, that was reconstructed and inserted by superimposition with GOT1. Note in particular Leu348 (in purple), whose side chain is pointing towards the methyl group of L-alanine.

References

1. Bifulco, D.; Pollegioni, L.; Tessaro, D.; Servi, S.; Molla, G. A thermostable L-aspartate oxidase: A new tool for biotechnological applications. *Appl. Microbiol. Biotechnol.* **2013**, *97*, 7285–7295.
2. Leese, C.; Fotheringham, I.; Escalettes, F.; Speight, R.; Grogan, G. Cloning, expression, characterisation and mutational analysis of L-aspartate oxidase from *Pseudomonas putida*. *J. Mol. Catal. B Enzym.* **2013**, *85–86*, 17–22.
3. Cardia, J.P.; Eldo, J.; Xia, J.; O'Day, E.M.; Tsuruta, H.; Gryncel, K.R.; Kantrowitz, E.R. Use of L-asparagine and N-phosphonacetyl-L-asparagine to investigate the linkage of catalysis and homotropic cooperativity in *E. coli* aspartate transcarbomoylase. *Proteins Struct. Funct. Genet.* **2008**, *71*, 1088–1096.
4. Keng, Y.; Viola, R.E. Specificity of aspartokinase III from *Escherichia coli* and an examination of important catalytic residues. *Arch. Biochem. Biophys.* **1996**, *335*, 73–81.
5. Wei, J.F.; Yang, H.W.; Wei, X.L.; Qiao, L.Y.; Wang, W.Y.; He, S.H. Purification, characterization and biological activities of the L-amino acid oxidase from *Bungarus fasciatus* snake venom. *Toxicon* **2009**, *54*, 262–271.
6. Tedeschi, G.; Negri, A.; Ceciliani, F.; Ronchi, S.; Vetere, A.; D'Aniello, G.; D'Aniello, A. Properties of the flavoenzyme D-aspartate oxidase from *Octopus vulgaris*. *Biochim. Biophys. Acta (BBA)/Protein Struct. Mol.* **1994**, *1207*, 217–222.
7. D'Aniello, A.; Vetere, A.; Petrucelli, L. Further study on the specificity of D-amino acid oxidase and of D-aspartate oxidase and time course for complete oxidation of D-amino acids. *Comp. Biochem. Physiol. -- Part B Biochem.* **1993**, *105*, 731–734.
8. Takahashi, S.; Osugi, K.; Shimekake, Y.; Shinbo, A.; Abe, K.; Kera, Y. Characterization and improvement of substrate-binding affinity of D-aspartate oxidase of the thermophilic fungus *Thermomyces dupontii*. *Appl. Microbiol. Biotechnol.* **2019**, *103*, 4053–4064.
9. Cronin, C.N.; Kirsch, J.F. Role of arginine-292 in the substrate specificity of aspartate aminotransferase as examined by site-directed mutagenesis. *Biochemistry* **1988**, *27*, 4572–4579.
10. Reczkowski, R.R.; Ash, D.E. Rat liver arginase: kinetic mechanism, alternate substrates and inhibitors. *Arch. Biochem. Biophys.* **1994**, *312*, 31–37.
11. Saidha, T.; Schiff, J.A. Purification and properties of a phenol sulphotransferase from *Euglena* using L-tyrosine as substrate. *Biochem. J.* **1994**, *298*, 45–50.
12. Genet, R.; Bénetti, P.H.; Hammadi, A.; Ménez, A. L-Tryptophan 2′,3′-oxidase from *Chromobacterium violaceum*: Substrate specificity and mechanistic implications. *J. Biol. Chem.* **1995**, *270*, 23540–23545.
13. Mashabela, G.T.M.; Seebeck, F.P. Substrate specificity of an oxygen dependent sulfoxide synthase in ovothiol biosynthesis. *Chem. Commun.* **2013**, *49*, 7714–7716.
14. Sorci, L.; Cimadamore, F.; Scotti, S.; Petrelli, R.; Cappellacci, L.; Franchetti, P.; Orsomando, G.; Magni, G. Initial-rate kinetics of human NMN-adenylyltransferases: Substrate and metal ion specificity, inhibition by products and multisubstrate analogues, and isozyme contributions to NAD+ biosynthesis. *Biochemistry* **2007**, *46*, 4912–4922.

15. Olland, A.M.; Underwood, K.W.; Czerwinski, R.M.; Lo, M.C.; Aulabaugh, A.; Bard, J.; Stahl, M.L.; Somers, W.S.; Sullivan, F.X.; Chopra, R. Identification, characterization, and crystal structure of *Bacillus subtilis* nicotinic acid mononucleotide adenylyltransferase. *J. Biol. Chem.* **2002**, *277*, 3698–3707.
16. Vergauwen, B.; Pauwels, F.; Jacquemotte, F.; Meyer, T.E.; Cusanovich, M.A.; Bartsch, R.G.; Van Beeumen, J.J. Characterization of glutathione amide reductase from *Chromatium gracile*. Identification of a novel thiol peroxidase (Prx/Grx) fueled by glutathione amide redox cycling. *J. Biol. Chem.* **2001**, *276*, 20890–20897.
17. Chang, A.; Jeske, L.; Ulbrich, S.; Hofmann, J.; Koblitz, J.; Schomburg, I.; Neumann-Schaal, M.; Jahn, D.; Schomburg, D. BRENDA, the ELIXIR core data resource in 2021: New developments and updates. *Nucleic Acids Res.* **2021**, *49*, D498–D508.
18. Ueno, Y.; Hayakawa, K.; Takahashi, S.; Oda, K. Purification and charachterization of glutamate decarboxylase from *Lactobacillus brevis* IFO 12005. *Biosci. Biotechnol. Biochem.* **1997**, *61*, 1168–1171.
19. Vacca, R.A.; Giannattasio, S.; Graber, R.; Sandmeier, E.; Marra, E.; Christen, P. Active-site Arg→Lys substitutions alter reaction and substrate specificity of aspartate aminotransferase. *J. Biol. Chem.* **1997**, *272*, 21932–21937.
20. Kato, S.; Ikuta, T.; Hemmi, H.; Takahashi, S.; Kera, Y.; Yoshimura, T. Enzymatic assay for D-aspartic acid using D-aspartate oxidase and oxaloacetate decarboxylase. *Biosci. Biotechnol. Biochem.* **2012**, *76*, 2150–2152.
21. Uchida, Y.; Hayashi, H.; Washio, T.; Yamasaki, R.; Kato, S.; Oikawa, T. Cloning and characterization of a novel fold-type I branched-chain amino acid aminotransferase from the hyperthermophilic archaeon *Thermococcus sp.* CKU-1. *Extremophiles* **2014**, *18*, 589–602.
22. Rodríguez, S.B.; Stitt, B.L.; Ash, D.E. Expression of peptidylarginine deiminase from *Porphyromonas gingivalis* in Escherichia coli: Enzyme purification and characterization. *Arch. Biochem. Biophys.* **2009**, *488*, 14–22.
23. Bhatia, M.B.; Grubmeyer, C. The role of divalent Magnesium in activating the reaction catalyzed by orotate phosphoribosyltransferase. *Arch. Biochem. Biophys.* 1993, *303*, 321–325.
24. Born, T.L.; Franklin, M.; Blanchard, J.S. Enzyme-catalyzed acylation of homoserine: Mechanistic characterization of the *Haemophilus influenzae* met2-encoded homoserine transacetylase. *Biochemistry* **2000**, *39*, 8556–8564.
25. Suzuki, H.; Nakayama, T.; Nagae, S.; Yamaguchi, M.A.; Iwashita, T.; Fukui, Y.; Nishino, T. cDNA cloning and functional characterization of flavonol 3-O-glucoside-6″-O-malonyltransferases from flowers of *Verbena hybrida* and *Lamium purpureum*. *J. Mol. Catal. B Enzym.* **2004**, *28*, 87–93.
26. Ohshima, T.; Nishida, N.; Bakhtavasalam, S.; Kataoka, K.; Takada, H.; Yoshimura, T.; Esaki, N.; Soda, K. The purification, characterization, cloning and sequencing of the gene for a halostable and thermostable leucine dehydrogenase from *Thermoactinomyces intermedius*. *Eur. J. Biochem.* **1994**, *222*, 305–312.
27. Xing, R.; Whitman, W.B. Characterization of amino acid aminotransferases of *Methanococcus aeolicus*. *J. Bacteriol.* **1992**, *174*, 541–548.
28. Maloney, G.S.; Kochevenko, A.; Tieman, D.M.; Tohge, T.; Krieger, U.; Zamir, D.; Taylor, M.G.; Fernie, A.R.; Klee, H.J. Characterization of the branched-chain amino acid aminotransferase enzyme family in tomato. *Plant Physiol.* **2010**, *153*, 925–936.
29. Tawfik, D.S.; Gruic-Sovulj, I. How evolution shapes enzyme selectivity – lessons from aminoacyl-tRNA synthetases and other amino acid utilizing enzymes. *FEBS J.* **2020**, *287*, 1284–1305.
30. Martin, D.L. Regulatory properties of brain glutamate decarboxylase. *Cell. Mol. Neurobiol.* **1987**, *7*, 237–253.
31. Klein, M.; Kaltwasser, H.; Jahns, T. Isolation of a novel, phosphate-activated glutaminase from *Bacillus pasteurii*. *FEMS Microbiol. Lett.* **2002**, *206*, 63–67.
32. Reda, F.M. Kinetic properties of *Streptomyces canarius* L- Glutaminase and its anticancer efficiency. *Brazilian J. Microbiol.* **2015**, *46*, 957–968.
33. Roberts, J. Purification and properties of a highly potent antitumor glutaminase asparaginase from Pseudomonas 7A. *J. Biol. Chem.* **1976**, *251*, 2119–2123.
34. Guranowski, A.; Jakubowski, H. Adenosylhomocysteinase from lellow lupine. *Methods Enzymol.* **1987**, *143*, 430–434.
35. Matoba, Y.; Yoshida, T.; Izuhara-Kihara, H.; Noda, M.; Sugiyama, M. Crystallographic and mutational analyses of cystathionine β-synthase in the H2S-synthetic gene cluster in *Lactobacillus plantarum*. *Protein Sci.* **2017**, *26*, 763–783.
36. Majtan, T.; Krijt, J.; Sokolová, J.; Křížková, M.; Ralat, M.A.; Kent, J.; Gregory, J.F.; Kožich, V.; Kraus, J.P. Biogenesis of hydrogen sulfide and thioethers by cystathionine beta-synthase. *Antioxidants Redox Signal.* **2018**, *28*, 311–323.
37. El-Sayed, A.S.; Khalaf, S.A.; Aziz, H.A. Characterization of homocysteine γ-lyase from submerged and solid cultures of *Aspergillus fumigatus* ASH (JX006238). *J. Microbiol. Biotechnol.* **2013**, *23*, 499–510.
38. Kusakabe, H.; Kodama, K.; Kuninaka, A.; Yoshino, H.; Misono, H.; Soda, K. A new antitumor enzyme , L-lysine α-oxidase from *Trichoderma viride*. *J. Biol. Chem.* **1980**, *255*, 976–981.
39. Nakazawa, T.; Hori, K.; Hayashi, O. Studies on Monooxygenases. V. Manifestation of amino acid oxidase activity by lysine monooxygenase. *J. Biol* **1972**, *247*, 3439–3444.
40. Kato, S.; Hemmi, H.; Yoshimura, T. Lysine racemase from a lactic acid bacterium, *Oenococcus oeni*: Structural basis of substrate specificity. *J. Biochem.* **2012**, *152*, 505–508.
41. Lienhard, G.E. Enzymatic catalysis and transition-state theory. *Science.* **1973**, *180*, 149–154.
42. Fersht, A. Structure and mechanism in protein science: A guide to enzyme catalysis and protein folding.; W H Freeman & Co, New York, NY, USA, 1999.
